# Supplementary material for: Genome Characterization and Spaciotemporal Dispersal Analysis of Bagaza Virus Detected in Portugal, 2021
Source: Pathogens. 2023 Jan 17;12(2):150. doi: 10.3390/pathogens12020150 (PMC9962176; doi:10.3390/pathogens12020150)
Supplement: Supplementary file 1 [file pathogens-12-00150-s001.zip › Supplementary_Table_S1_Falcão_et_al.pdf]

Supplementary materials

Table S1. Referenced viruses strains used in this study.

No	Virus	GenBank Nº	Year	Host	Location
**1**	BAGV	LC730845	2021	Red-legged partridge	Portugal
**2**	BAGV	HQ644144	2010	Red-legged partridge	Spain
**3**	BAGV	KR108245	2010	Red-legged partridge	Spain
**4**	BAGV	KR108246	2010	Red-legged partridge	Spain
**5**	BAGV	KR108244	2010	Red-legged partridge	Spain
**6**	BAGV	HQ644143	2010	Red-legged partridge	Spain
**7**	BAGV	LC318701	2013	Culex quinquefasciatus	Zambia
**8**	BAGV	MW672101	2018	Culex univittatus	Namibia
**9**	BAGV	MF380429	1988	Culex poicilipes	Cote d'Ivoire
**10**	BAGV	MF380430	1989	Culex poicilipes	Senegal
**11**	BAGV	MF380434	2014	Culex neavei	Senegal
**12**	BAGV	MF380433	2001	Culex poicilipes	Senegal
**13**	BAGV	MF380428	2004	Culex poicilipes	Senegal
**14**	BAGV	MF380426	2000	Culex poicilipes	Senegal
**15**	BAGV	MF380432	2001	Culex poicilipes	Senegal
**16**	BAGV	MF380431	1994	Culex neavei	Senegal
**17**	BAGV	MF380427	2004	Culex poicilipes	Senegal
**18**	BAGV	MF380425	2000	Culex poicilipes	Senegal
**19**	BAGV	MF380424	1989	Culex poicilipes	Senegal
**20**	BAGV	NC012534	NA	Mosquito	Central African Republic
**21**	BAGV	EU684972	1996	Culex tritaeniorhynchus	India

**22**	ITMV	KC734552	2010	Turkey	Israel
**23**	ITMV	KC734550	1995	Turkey	Israel
**24**	ITMV	KC734549	1985	Turkey	Israel
**25**	ITMV	KF917537	1959	Turkey	Israel
**26**	TMUV	JX477685	1955	NA	Malaysia
**27**	TMUV*	JX477686	2000	NA	Malaysia
**28**	TMUV	MW821486	2020	Goose	Taiwan
**29**	TMUV	MW922032	2019	Mallard	Taiwan
**30**	TMUV	MH748542	2014	Mallard	China
**31**	TMUV	KT607936	2012	Culex tritaeniorhynchus	China
**32**	TMUV	KT607935	2012	Culex tritaeniorhynchus	China
**33**	TMUV	KX097990	2012	Pekin duck	Malaysia
**34**	TMUV	MF621927	2007	Duck	Thailand
**35**	TMUV	KR061333	2013	Duck	Thailand
**36**	TMUV	MH460536	2015	Mosquito	Thailand
**37**	TMUV	MT708901	2019	Goose	China
**38**	TMUV	MN966679	2019	Duck	China
**39**	TMUV	MT951413	2020	Goose	China
**40**	TMUV	MT951412	2020	Duck	China
**41**	TMUV	KY623436	2015	Duck	China
**42**	TMUV	KX686579	2015	layer duck	China
**43**	TMUV	KM275941	2013	Duckling	China
**44**	TMUV	KJ782380	2012	Chicken	China
**45**	TMUV*	JF926699	2010	Layer duck	China
**46**	TMUV	KP096415	2014	Duck	China
**47**	TMUV	MT447092	2018	Duck	China
**48**	TMUV	KF826767	2012	House Sparrow	China
**49**	TMUV	MZ574097	2019	Duck	China
**50**	TMUV	KY810818	2015	Duck	China
**51**	TMUV	KX452096	2014	Duck	China
**52**	TMUV	KJ740745	2013	Layer Duck	China

**53**	TMUV	MH764607	2017	Meat Ducks	China
**54**	TMUV	MH764606	2016	Meat Ducks	China
**55**	TMUV	JQ289550	2010	Duck	China
**56**	TMUV*	JQ920425	2010	Pigeon	China
**57**	TMUV	MN649267	2014	Duck	China
**58**	TMUV	KC990540	2010	Duck	China
**59**	TMUV	KC990541	2012	Duck	China
**60**	NTAV	KF917539	1943	Mosquito	Uganda
**61**	NTAV	NC018705	1966	NA	Cameroon
**62**	Rocio	NC040776	NA	NA	NA
**63**	ILHV	MH932545	1994	Anopheles triannulatus	Brazil
**64**	ILHV	NC009028	NA	NA	NA
**65**	ILHV	KC481679	2010	Aedes scapularis	Brazil
**66**	Entebbe bat	KP233893	2011	Little free-tailed bat	Uganda
**67**	Sokoluk	KF917541	1970	Pipistrellus	Kyrgyztam
**68**	Yokose	MH051229	2013	Daubenton's bat	China
**69**	Montana myotis leukoencephalitis	AJ299445	NA	NA	USA
**70**	Rio Bravo	AF144692	NA	NA	USA
**71**	Jutiaba	KJ469371	1969	Hispid cotton rat	Guatemala
**72**	Modoc	AJ242982	NA	NA	USA
**73**	Cell fusing agent	NC001564	2012	Aedes aegypti	USA
**74**	Aedes flavivirus	AB488408	2003	Aedes albopictus	Japan
**75**	Palm Creek	KC505248	2010	Coquillettidia xanthogaster	Australia
**76**	Culex flavivirus	GQ165808	2008	Culex quinquefasciatus	Uganda
**77**	Culex theileri flavivirus	HE574573	2011	Culex theileri	Portugal
**78**	Tyuleniy	KT224356	1986	Seabird Tick	Russia
**79**	Karshi	DQ462443	1978	Ornithodoros tick	Turkmenistan
**80**	Royal Farm	NC039219	NA	NA	Afghanistan

**81**	Meaban	DQ235144	NA	NA	France
**82**	Langat	MK680893	1973	Haemaphysalis tick	Thailand
**83**	Powassan	MZ576219	2020	Hard-bodied tick	USA
**84**	Alkhumra	NC004355	1995	Human	Saudi Arabia
**85**	Tick borne encephalitis	JN003208	2002	Human	Russia
**86**	Louping ill	MN844186	1991	Sheep	UK
**87**	Bouboui	DQ859057	NA	NA	Central African Republic
**88**	Banzi	DQ859056	NA	NA	South Africa
**89**	Potiskum	DQ859067	NA	NA	Papua New Guinea
**90**	Wesselsbron	JN226796	1997	Human	South Africa
**91**	Yellow Fever	MF370533	2017	Human	Brazil
**92**	Lammi	FJ606789	2004	Mosquito	Finland
**93**	Chaoyang	FJ883471	2008	Mosquito	China
**94**	Ilomantsi	KC692067	2007	Mosquito	Finland
**95**	Donggang	NC016997	2009	Aedes sp.	China
**96**	Zika	KX893855	2016	Human	Venezuela
**97**	Dengue	AY618991	1977	NA	Thailand
**98**	Dengue	EU056812	1977	Human	Puerto Rico
**99**	Dengue	EF105385	1974	Aedes luteocephalus	Senegal
**100**	Dengue	AY099336	2000	NA	Sri Lanka
**101**	Dengue	EU482822	2006	Human	Vietnam
**102**	Iguape	MK332105	1994	Anopheles cruzii	Brazil
**103**	Usutu	AY453411	2001	Bird	Austria
**104**	Japanese encephalitis	LC705012	2012	Cattle	Japan
**105**	Koutango	MN057643	2016	Sandfly	Niger
**106**	Kunjin	KX394405	1963	Culex annulirostris	Australia
**107**	WNV	DQ786572	2004	House Sparrow	France
**108**	Baiyangdian	JF312912	2010	Duck	China

*Sitiawan virus, Baiyangdian virus and Duck egg-drop syndrome virus (DEDSV) are currently considered to be subtypes of TMUV.
