# Supplementary figures and images for: Genome Characterization and Spaciotemporal Dispersal Analysis of Bagaza Virus Detected in Portugal, 2021
Source: Pathogens. 2023 Jan 17;12(2):150. doi: 10.3390/pathogens12020150 (PMC9962176; doi:10.3390/pathogens12020150)

A

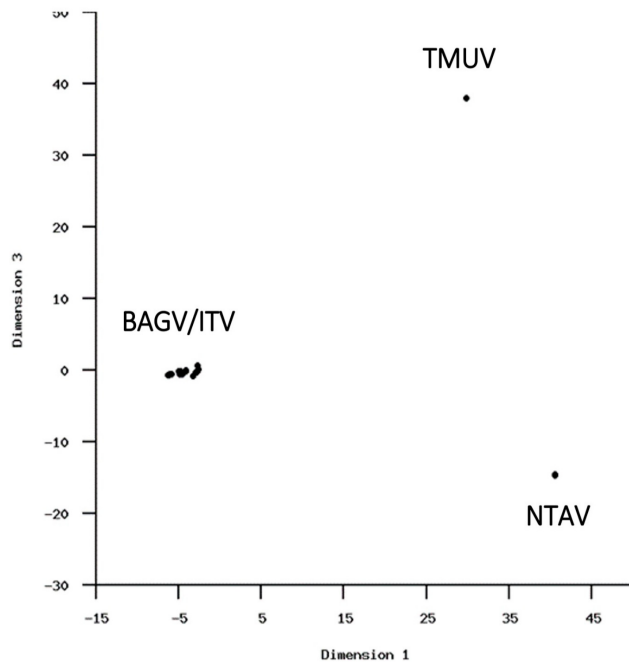

B

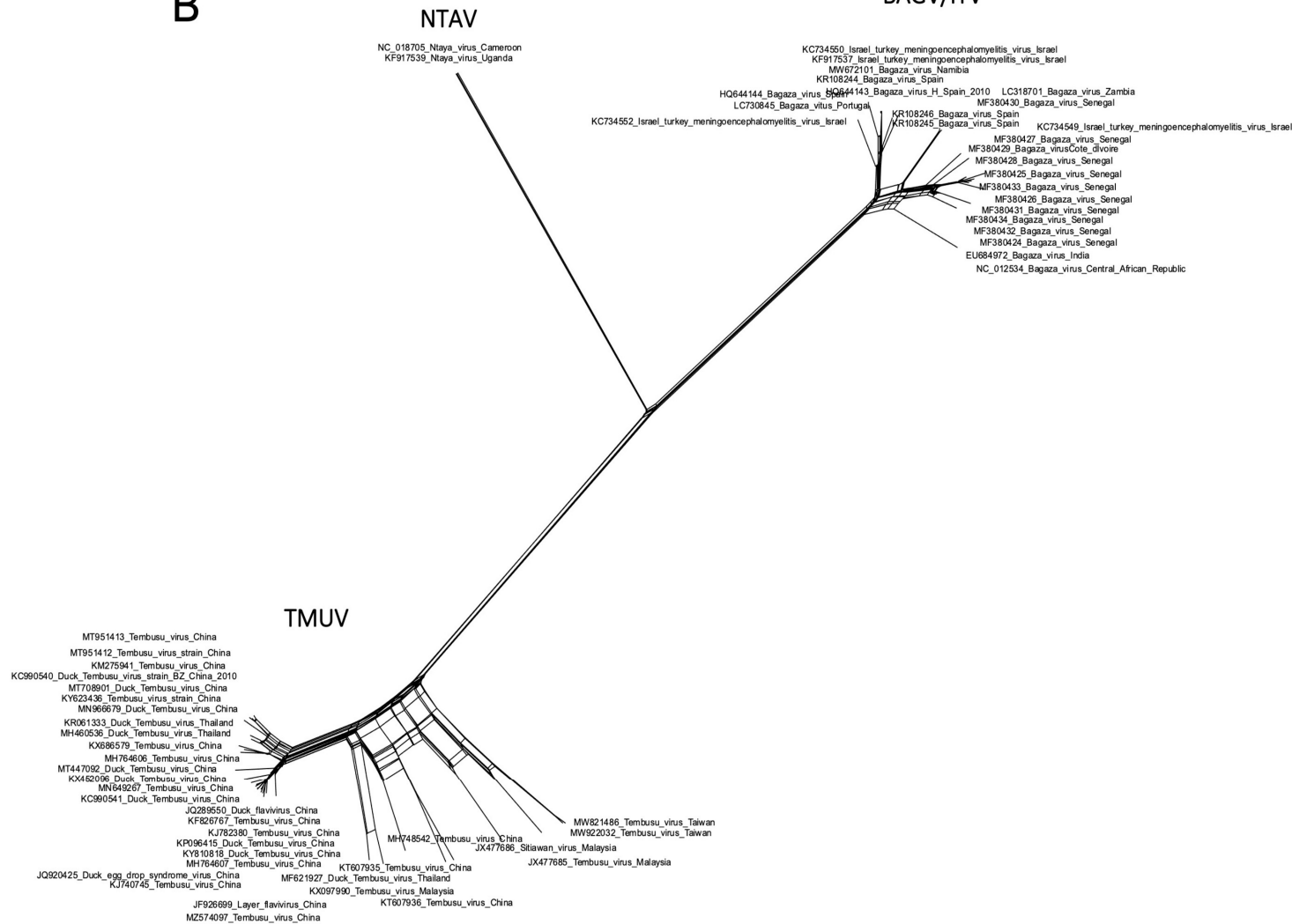

BAGV/ITV

Supplement: Supplementary file 1 [file pathogens-12-00150-s001.zip › Supplementary_Figure_S1_Falcão_et_al.pdf]
